# The Protective Effect and Mechanism of Dexmedetomidine on Diabetic Peripheral Neuropathy in Rats

**DOI:** 10.3389/fphar.2020.01139

**Published:** 2020-07-30

**Authors:** Yan-zhuo Zhang, Zhong-cheng Zhou, Chun-yu Song, Xia Chen

**Affiliations:** ^1^ Department of Anesthesiology, The Fourth Affiliated Hospital of Guangxi Medical University/Liuzhou Workers’ Hospital, Liuzhou, China; ^2^ Department of Anesthesiology, China and Heilongjiang Key Laboratory for Anesthesia and Critical Care, The Second Affiliated Hospital of Harbin Medical University, Harbin, China

**Keywords:** dexmedetomidine, diabetic peripheral neuropathy, protective effect, mechanism, rats

## Abstract

**Objective:**

To investigate the role of dexmedetomidine (DEX) in the inhibition of diabetic peripheral neuropathy (DPN) and the protection in the nerve damage.

**Methods:**

Eighty male Sprague-Dawley (SD) rats were randomly allocated to four groups: the control group (C group), DPN model group (DPN group), DEX-treated group (DEX group), and the yohimbine treated group (YOH group). DPN was induced by intraperitoneal administration of streptozocin (STZ) (35 mg/kg). The body weights, blood glucose level, mechanical withdrawal threshold (MWT), thermal withdrawal latency (TWL), the motor, and sensory nerve conduction velocities (MNCV and SNCV) of sciatic nerve were measured. Then the sciatic nerve was isolated for H&E staining and immunohistochemical staining. The oxidative stress makers such as malondialdehyde (MDA), superoxide-dismutase (SOD), and glutathione peroxidase (GSH-Px) and apoptosis related cytokines such as Bax, Bcl-2, and caspase-3 were estimated.

**Results:**

There was no significant difference of the blood glucose and body weight among the DPN group, DEX group, and YOH group. H&E staining showed that DEX treatment can ameliorate the damage of sciatic nerve cells. In the DPN group, MWT, TWL, MNCV, and SNCV were significantly reduced compared with the C group (P < 0.05). In DEX group rats, MWT, TWL, MNCV, and SNCV were increased significantly (P < 0.05) compared with the DPN group and YOH group rats. Lower SOD and GSH-Px, and higher MDA were found in the DPN group compared with the C group (P < 0.01), and DEX treatment restored SOD, GSH-px, and MDA activity significantly (P < 0.01). The expression levels of Bax and caspase-3 were increased, while that of Bcl-2 was decreased significantly in the DPN group compared with the C group (P < 0.05). In the DEX group, the expression levels of Bax and caspase-3 were decreased significantly (P < 0.05), while that of Bcl-2 was increased significantly (P < 0.05) compared with the DPN group and the YOH group.

**Conclusion:**

The results of this study demonstrated that DEX has the inhibitory and protective effects on DPN of rats. This may be associated with its antioxidative and anti-apoptosis responses.

## Introduction 

Diabetic neuropathic pain is one of the most serious complications of diabetes mellitus (DM), which will afflict the quality of the patients’ life (Javed et al., 2019). Generally, neuropathy develops over time, thus measures need to be taken to protect the nervous system from diabetes. Moreover, even when blood sugar is strictly controlled, nerve damage is often unavoidable (Harati, 2007). However, there are insufficient methods to prevent and treat such neuropathy and the pain (Howard and Charles, 2011). Because of its huge unmet needs and medical importance, diabetic peripheral neuropathy (DPN) is being studied in depth.

Recent work showed that hyperglycemia leads to excessive generation of superoxide anions in mitochondria and causes oxidative stress in tissue cells, which is a common mechanism leading to chronic complications of diabetes including DPN (Vincent et al., 2004; Ziegler et al., 2004). Stavniichuk (Stavniichuk et al., 2011) found that mitochondrial swelling, internal crest rupture, and typical apoptosis can change in the dorsal root ganglion (DRG) tissues of DM rats induced by streptozocin (STZ) at 12 weeks, and the expression of activated caspase-9 is increased. These mitochondrial changes were also observed in sural nerve biopsies from patients with DPN. Schmeichel (Schmeichel et al., 2003) demonstrated that superoxide anion level was increased, then caspase-3 activation was increased, and superoxide dismutase (SOD) 2 expression was decreased in the DRG in db/db diabetic mouse. This research indicated the critical role of apoptosis in the DPN. Oxidative stress can lead to cell apoptosis through the mitochondrial pathway, in which Cyt C initiates the enzyme cascade. Caspase-3 is the ultimate executor of apoptosis, and bcl-2 is a crucial anti-apoptotic regulator. Oxidative stress regulating the apoptosis is considered to be an important factor in the occurrence of neuropathy.

Dexmedetomidine (DEX) is a highly selective agonist of α_2_-receptors with sedative, anxiolytic, analgesic, and anesthetic properties. Recent studies have indicated that DEX exerts several non-anesthetic effects including antioxidant and immunomodulatory ones (Dong et al., 2014; Cakir et al., 2015; Wang et al., 2015). Furthermore, DEX has been demonstrated to have neuroprotective effect (Sifringer et al., 2015; Cheng et al., 2018; Li et al., 2018). In this study, firstly, the relationship between the development of diabetes and the onset of DPN was developed; secondly, the oxidative stress related malonaldehyde (MDA), SOD, glutathione (GSH), and apoptosis related proteins and genes Bax, Bcl-2, and caspase-3 in serum and sciatic nerve of rats induced DPN was investigated; finally, the protective and inhibitory effects of DEX on the DPN and the risk factors were evaluated.

## Materials and Methods

### Schematic Design of the Study

**Figure 1:**
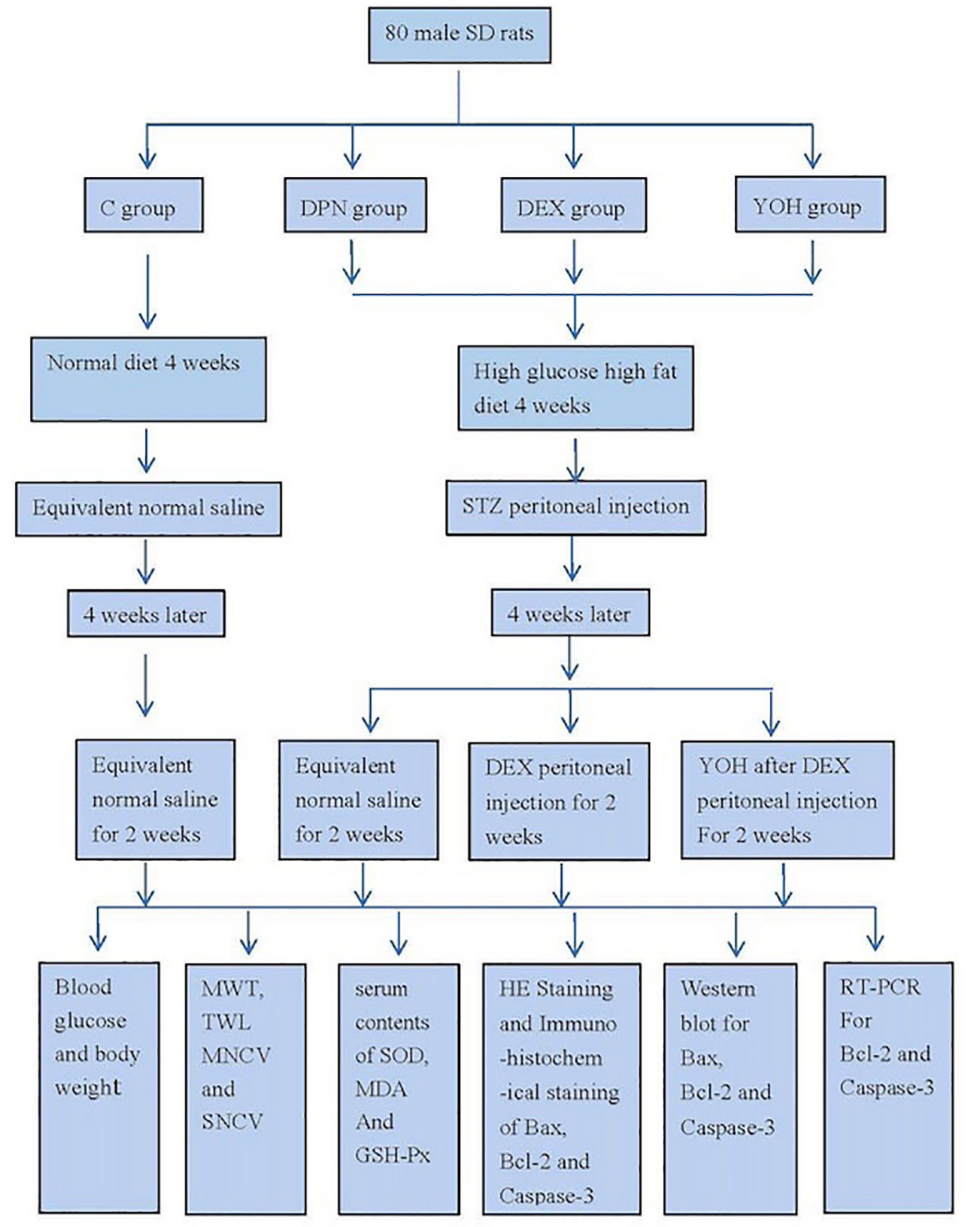
Schematic design of the study.

We carried out our research according to the schematic design of study (**Figure 1**).

#### Animal Preparation

The male Sprague-Dawley (SD) rats, weighing 80–100 g, were obtained from the animal research center of the Second Affiliated Hospital of Harbin Medical University. The study protocol was approved by Harbin Medical University’s Animal Ethics Committee, Harbin, China.

Eighty rats were randomly allocated to four groups (20 rats in each group): C group, DPN group, DEX group, and YOH group (treated with yohimbine). Yohimbine is a selective antagonist that blocks *α*_2_-adrenoceptor excitation of DEX. The rats were placed in plastic boxes at a controlled room temperature of 23–25 °C individually. The rats of the C group were given regular diet, but those of others three groups were given diets with high sugar and fat contents for 4 weeks. All of the rats’ blood glucose levels and body weights were monitored weekly

On the first day of fifth week, food was removed from rats for 16 h while removing water for 4 h for all groups before injection. In the DPN group, DEX group, and YOH group, the rats were injected with a dose of 35 mg/kg of STZ (Sigma-Aldrich, USA) once a day for two consecutive days intraperitoneally, whereas in the C group the rats were injected with the same capacity saline. On the second day of fifth week, the level of fasting glucose in the blood sample from the rats’ tail vein was measured. When the rat’s blood glucose level was not less than 16.7 mmol/L, DM model was confirmed. On the first day of every week before STZ injection, the mechanical withdrawal threshold (MWT) and the thermal withdrawal latency (TWL) were measured as the baseline. After the STZ injection, the MWT was calculated every 2 days, and an MWT less than 4 g is considered an indicator of successful DPN model. The rats of the DEX group and YOH group were injected with a dose of 25 μg/kg DEX (Hengrui Medicine, Jiangsu, China) once a day for 14 d from the ninth week intraperitoneally. In the YOH group, the rats were injected with 0.1 mg/kg yohimbine (Sigma-Aldrich, USA) 30 min after the DEX administration once a day for 14 d intraperitoneally.

#### Behavioral Tests

In the behavioral tests, MWT was measured as the mechanical stimulation response and TWL as the thermal stimulation response. For the MWT measurement, the rats were placed on a wire mesh platform and covered with a transparent glass container for 30 min for habituation. Then the rats were placed in the Plexiglas chamber and the rats’ plantar surface of the left hind paw was stimulated with the von Frey filaments (Stoeltin, USA). Each stimulation lasted for 3–5 s. A quick hind paw withdrawal was defined as a positive response. A force of 0.6 g was applied at the beginning, followed by ascending or descending consecutive stimuli according to the response of the rats; the force was then reduced after a positive response and the force was increased after a negative response. The minimal threshold force was determined when the withdrawal response was finally induced. It is noteworthy that to avoid tissue damage 15 g was set as the upper limit of force. Fifty percent of withdrawal threshold was obtained from the average MWT values.

The rats’ TWL was measured with the BME-410C full-automatic plantar analgesia tester (Youer Equipment Scientific Co., Ltd., Shanghai, China). The rats were placed on the surface of a 3 mm-thick glass plate covered with a Plexiglas chamber. And the heat stimuli were directed below the glass at the exposure site on the left hind paw. TWL was defined as the elapsed time to withdraw the paw. The average latency was calculated from the delivery of five thermal stimuli at 5 min intervals. A cut-off time of 60 s was set to avoid tissue damage (Lu et al., 2017).

#### Sciatic Nerve Conduction Velocity

The rats were anesthetized with intraperitoneal injection of 4 ml/kg 1% pentobarbital sodium. Then the skin was incised and the muscles were separated to expose the sciatic nerve, and then the stimulating and recording electrodes were directly placed under the sciatic nerve of the right leg. The stimulation electrode was set at the sciatic nerve notch and the recording electrode was put 6 mm far from it. The sciatic nerve was stimulated with a single square wave pulse (1.2 V in intensity, 1 ms in width) with the Functional Experiment System (BL-420 s, Taimeng, Sichuan, China). The motor nerve conductive velocity [MNCV (m/s) = D/L] with the action potential latency (L) of the sciatic nerve and the distance (D) between the stimulating and recording electrodes were calculated. For the sensory nerve conductive velocity (SNCV), the recording electrode was put in the sciatic notch and SNCV was calculated the same with the MNCV.

#### Oxidative Stress

To evaluate the oxidative stress, the serum contents of SOD, glutathione peroxidase (GSH-Px) and MDA. SOD activity was assessed with the xanthine oxidase method and the absorbance value was measured at 550 nm with a SOD kit (Jiancheng Bioengineering, Nanjing, China). MDA content was assessed with thiobarbituric acid reactive substances assay (Jiancheng Bioengineering, Nanjing, China) and the absorbance value was measured at a wavelength of 532 nm. GSH-Px activity was estimated by the analysis of glutathione in the enzymatic reaction. GSH-Px catalyzed the reduction of GSH in the presence of hydrogen peroxide. One unit of enzyme activity represents a decrease in GSH concentration of 1 μm/ml of serum (Yang et al., 2015).

#### Tissue Extraction and Preparation

On the 71th day, 4 ml/kg 1% pentobarbital sodium was intraperitoneally injected for the rats’ anesthesia and the rats’ hearts were perfused with 250 ml normal saline. The spinal cord was promptly placed on ice in a glass petri dish, the dorsal half of the lumbar cord was dissected and the sciatic nerves were isolated immediately. Eight tissue samples per group were frozen immediately in liquid nitrogen, stored at −70°C until assay for western-blot and Rt-PCR. The other tissues were kept in 4% paraformaldehyde for 24 h and embedded in paraffin for routine histological preparation. Then the paraffin-embedded tissue blocks horizontally were sectioned into 5 μm slices with a microtome and mounted them on poly-lysine-coated slides for hematoxyline–eosin (H&E) staining.

#### Immunohistochemical Staining of Bax, Bcl-2, and Caspase-3

The samples were prepared as described above and the sections were blocked in 10% normal goat serum at room temperature for 1 h. The cleaved Bax/Bcl-2 rabbit monoclonal antibody (1:200, Cell Signaling Technology Inc., Danvers, MA, USA) was diluted, which recognizes endogenous levels of the large fragment of activated Bax, and Bcl-2 rabbit monoclonal antibody in the recommended antibody diluents, then added to each section and incubated at 4°C overnight. The sections were rinsed three times in PBS and incubated with a biotinylated goat anti-rabbit secondary antibody (1:2,000) at room temperature for 1 h, then incubated with avidin-biotin-peroxidase solution (ABC reagent) for 30 min at room temperature, and washed three times in PBS. Then, the samples were treated with DAB for 2 min. Finally, the sections with hematoxylin, dehydrated and coverslipped were counterstained. The immunohistochemical staining of caspase-3 was the same as the Bax and Bcl-2 (Xi et al., 2011).

#### Western Blot Analysis for Bax, Bcl-2, and Caspase-3

The rats were sacrificed at 6 weeks after STZ injection. The dorsal root ganglion samples were dissolved by sonication for 5 s on ice in 0.5 ml of lysis buffer (50 mM Tris-Cl, pH 7.5, 2 mM EDTA, 100 mM NaCl, 1% Nonidet P-40, supplemented with protease inhibitor cocktail purchased from Sigma). Then the solubilized tissues were centrifuged at 12,000 rpm for 5 min at 4°C, and the supernatants containing protein were collected for immunoblot analysis. An equivalent amount of total protein (20–30 μg) was separated by sodium dodecyl sulfate polyacrylamide gel electrophoresis (SDS–PAGE) on 10% polyacrylamide gels and then transferred to polyvinylidine membranes. The membranes were blocked with 5% dry milk in PBS/0.1% (v/v) Tween 20 (PBST). Then the membranes were incubated with primary antibodies (anti-Bcl-2, anti-BAX, anti-caspase-3, and anti-actin, all from Santa Cruz) diluted in 2% BSA in PBST overnight at 4°C, washed three times with PBST (10 min each time), incubated with the appropriate horseradish peroxidase-conjugated secondary antibody for 45 min at room temperature, and then washed three times with PBST. Immunoreactive bands were visualized by enhanced chemiluminescence (ECL, Amersham Biosciences) with a standard X-ray film (Xi et al., 2011).

#### RT PCR Analysis

A moderate amount of dorsal root ganglion tissues of rats were rapidly transferred into 1 ml TRIzol reagent and fully ground into homogenate. The homogenate was put still at room temperature for 5 min until the sample was completely lysed. Then, centrifugation was performed at 12,000 g for 5 min at 4°C, and the supernatant was collected carefully, added with chloroform, mixed evenly, and placed still at room temperature for 5 min, followed by centrifugation at 12,000 g for 5 min at 4°C. After that, collected the supernatant carefully, added with the same volume of isopropanol, placed still at room temperature for 10 min, and centrifuged at 12,000 g for 10 min at 4°C. The precipitate was taken, added with 75% ethanol and mixed evenly; the RNA precipitation was washed. Later, added ribonuclease free (RNase-free) water to dissolve it completely. After that, the RNA concentration was measured with the ratio of the optical density at 260 and 280 nm (OD260/OD280). Last, according to the instructions, the primer sequence templates were amplified stepwise as shown in **Table 1**, and the reaction product was analyzed with reverse transcriptase-polymerase chain reaction (RT-PCR).

**Table 1:** RT-PCR primer sequences of Bcl-2, caspase-3, and β-actin messenger ribonucleic acid (mRNA).

Gene	Primer sequence	Amplified fragment length
Bcl-2	Upstream 5’-TGACTTCTCTCGTCGCTACC-3’Downstream 5’-GGTGACATCTCCCTGTTGAC-3’	198 bp
Caspase-3	Upstream 5’-GGACCTGTGGACCTGAAAAA-3’Downstream 5’-GCATGCCATATCATCGTCAG-3’	158 bp
β-actin	Upstream 5’-CCCATCTATGAGGGTTACGC-3’Downstream 5’-TTTAATGTCACGCACGATTTC-3’	150 bp

### Statistical Analysis

The results were presented as mean ± SEM and analyzed using SPSS 22.0. Statistical significance was analyzed by repeated measure analysis of variance, two-way ANOVA and two-tailed Student t-test, followed by *post-hoc* Tukey’s multiple comparison test. P values less than 0.05 were designated as statistically significant.

## Results

### The Effects of DEX on Serum Glucose and Body Weight

Before the induction of diabetes by STZ, there was no significant difference in the plasma glucose level between each group. After the injection of STZ, if the blood glucose exceeded 16.7 mmol/l, the rats were considered to have DM. All 60 rats eventually developed DM within 3 d after STZ injection. The serum glucose concentrations were increased significantly in the DPN group (21.51 ± 0.81 mmol/l), DEX group (22.28 ± 1.26 mmol/l), and YOH (21.80 ± 0.87 mmol/l) group compared with the C group (5.40 ± 0.23 mmol/l, p < 0.05). The blood glucose was also higher 6 weeks after STZ injection (22.95 ± 1.06 mmol/l in the DPN group, 22.09 ± 0.76 mmol/l in the DEX group, 21.54 ± 1.20 mmol/l in the the YOH group) than that (5.51 ± 0.37 mmol/l) in the C group (p < 0.05, **Table 2**). However, 25 μg/kg DEX had no significant effect on the blood glucose of the rats among the three groups. Our results also showed significant changes of weights. In the C group, the weights of the rats were increased over time. The weights of the rats in the DPN group, DEX group, and YOH group were increased significantly from 1 d to 35 d and were decreased significantly during 35–70 d (p < 0.05). At the end of the study (week 10), the weights of the rats in the DPN group, DEX group, and YOH group were significantly lower than that in the C group (p < 0.05, **Table 2**).

**Table 2:** The effect of dexmedetomidine on serum glucose levels and body weight (n=20, mean ± SEM).

Group	Serum glucose (mmol/l)			Body weight (g)		
	1 d	35 d	70 d	1 d	35 d	70 d
C	5.42 ± 0.25	5.40 ± 0.23	5.51 ± 0.37	150.12 ± 3.10	218.63 ± 4.23*	273.32 ± 8.58*^#^
DPN	5.50 ± 0.21	21.51 ± 0.81*^&^	22.95 ± 1.06*^&^	149.10 ± 2.87	213.32 ± 5.61*	176.12 ± 5.74*^#&^
DEX	5.43 ± 0.19	22.28 ± 1.26*^&^	22.09 ± 0.76*^&^	152.12 ± 2.98	214.23 ± 7.73*	180.13 ± 7.53*^#&^
YOH	5.44 ± 0.25	21.80 ± 0.87*^&^	21.54 ± 1.20*^&^	149.94 ± 3.01	216.19 ± 8.57*	187.24 ± 6.37*^#&^

C, control; DPN, diabetic peripheral neuropathy; DEX, dexmedetomidine; YOH, yohimbine.

*p < 0.05 compared with the 1 d at the same group, ^#^p < 0.05 compared with the 35 d at the same group, ^&^p < 0.05 compared with the C group on the same day.

### The Protective Effects of DEX Treatment on MWT and TWL

**Figure 2:**
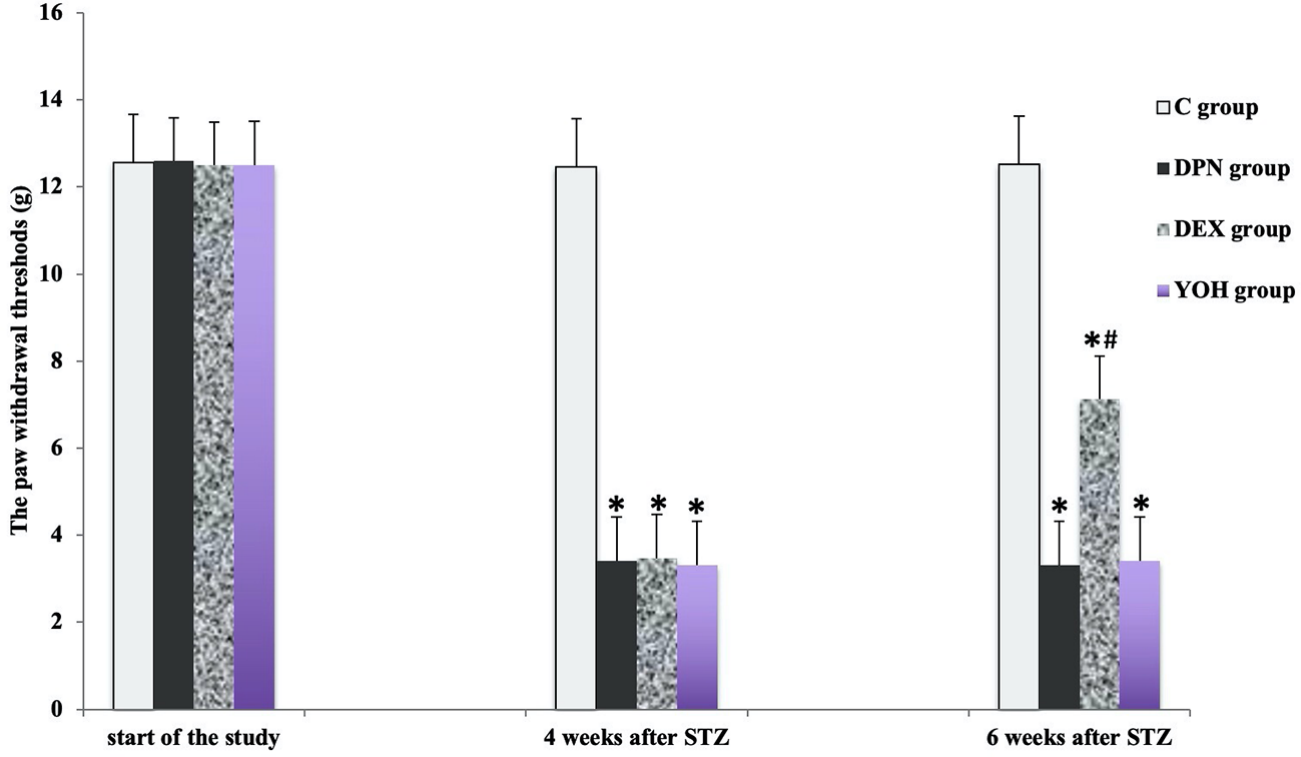
The mechanical withdraw threshold in each group [n = 20, *p < 0.05 compared with the control (C) group; ^#^p < 0.05 compared with the diabetic peripheral neuropathy (DPN) group].

**Figure 3:**
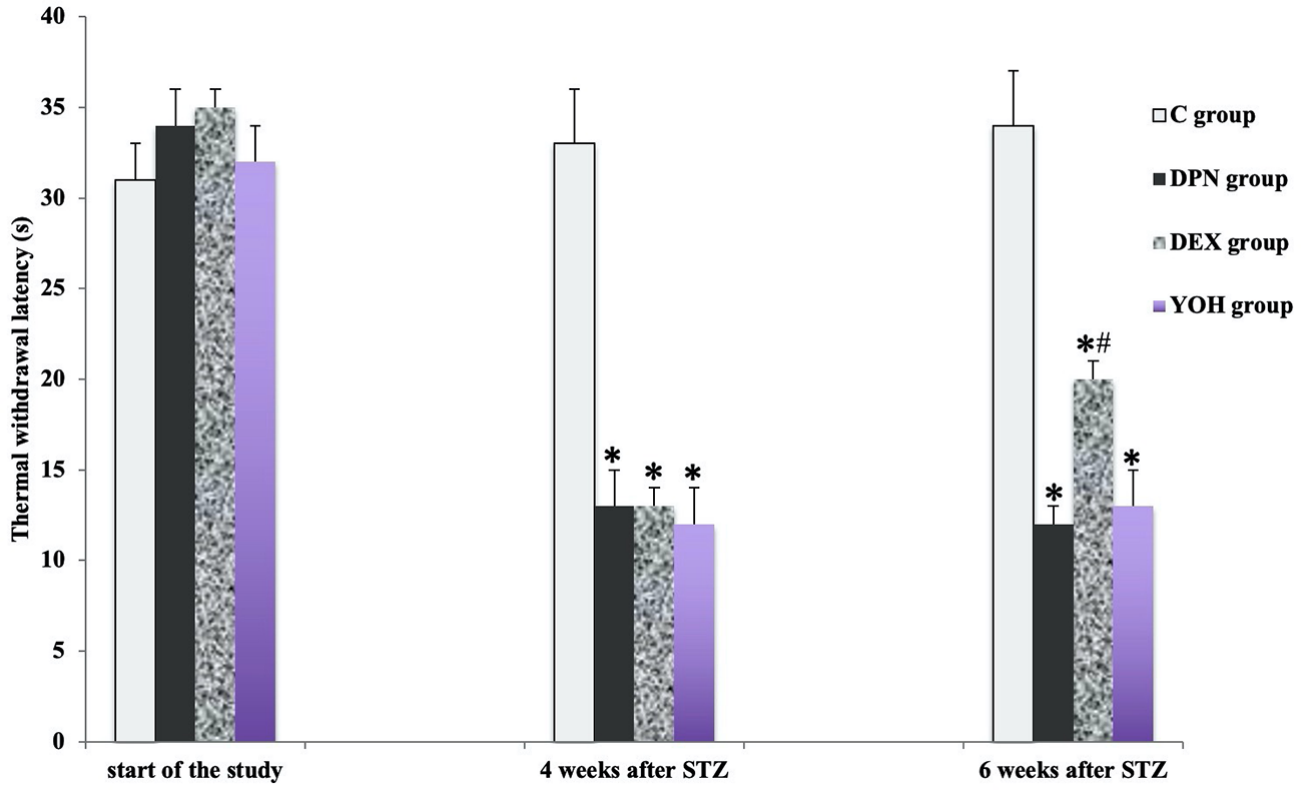
The thermal withdraw latency in each group [n = 20, *p < 0.05 compared with the control (C) group; ^#^p < 0.05 compared with diabetic peripheral neuropathy (DPN) group].

There was no significant difference in the mean MWT and TWL among all groups before the induction of diabetic neuropathy. At 4 weeks after STZ injection, the average MWT and TWL of the DPN group, the DEX group, and the YOH group were significantly reduced than that of the C group (p < 0.05). The mean paw withdrawal threshold was ameliorated significantly in the DEX group compared with that in the DPN group and YOH group (p < 0.05, **Figures 2** and **3**).

### Effect of DEX on Nerve Conduction Velocity

**Figure 4:**
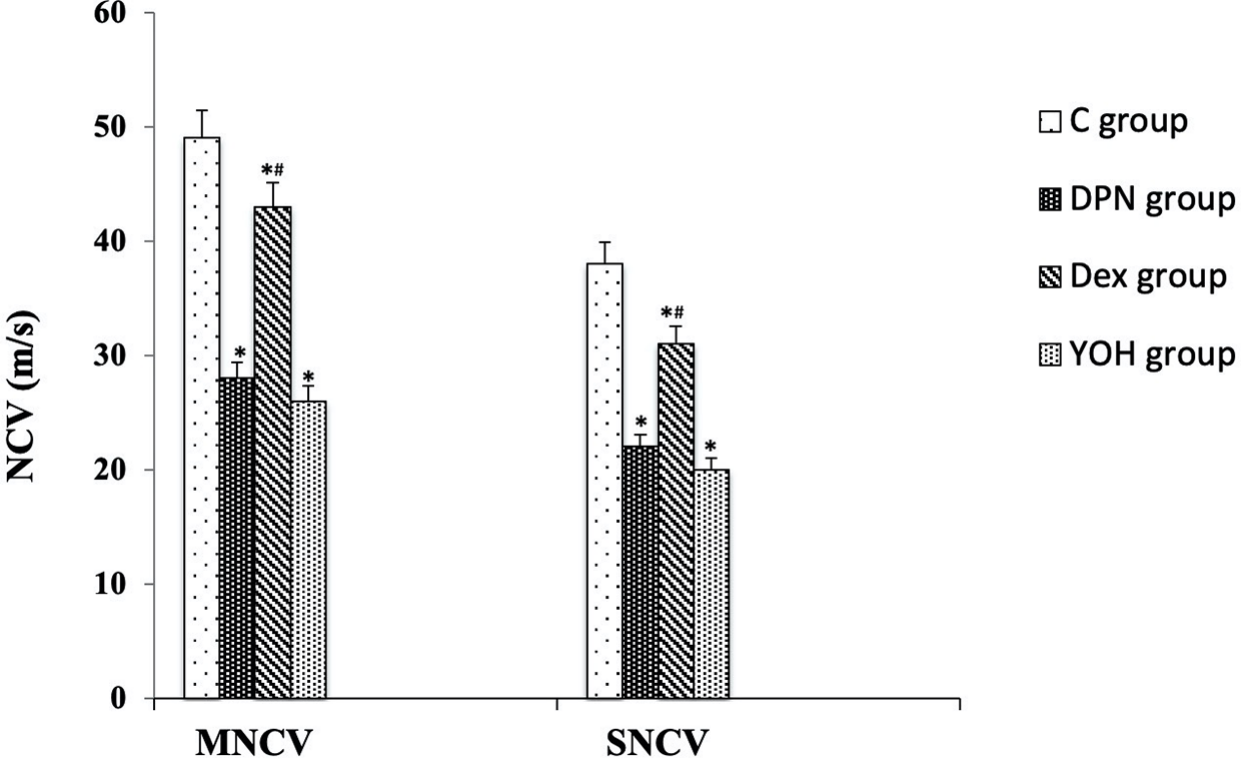
Motor and sensory nerve conduction velocities (MNCV and SNCV) in each group [n = 20, *p < 0.05 compared with the control (C) group; ^#^p < 0.05 compared with diabetic peripheral neuropathy (DPN) group].

In the DPN group, both MNCV and SNCV were significantly lower than those in the C group (p < 0.01). In DEX treated DPN rats, both MNCV and SNCV were significantly higher than those in the DPN group and YOH group (p < 0.01, **Figure 4**).

### Effect of DEX on Oxidative Stress

**Figure 5:**
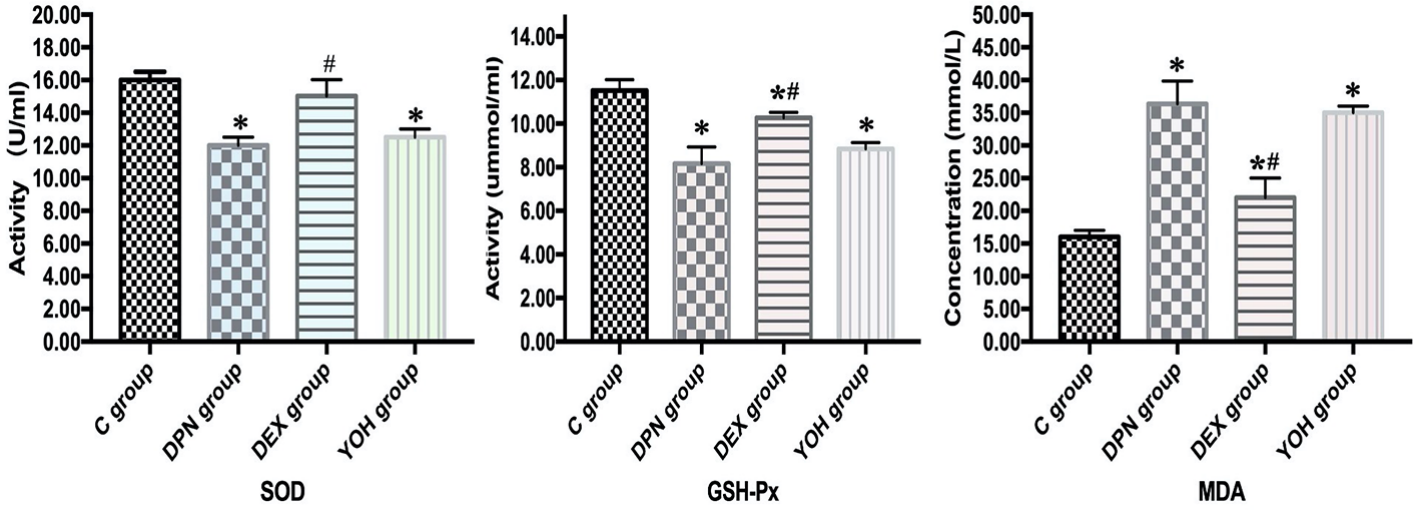
Oxidative stress parameters in each group [n = 20, *p < 0.05 compared with the control (C) group; ^#^p < 0.05 compared with diabetic peripheral neuropathy (DPN) group].

In the DPN group, the serum SOD and GSH-Px were significantly lower than those in the C group (p < 0.01), and in the DEX group, DEX administration reversed SOD and GSH-Px activity significantly (p < 0.01). The serum contents of MDA in the DPN group were significantly higher than those in the C group (p < 0.01), and DEX treatment decreased the serum contents of MDA significantly (p < 0.01, **Figure 5**).

### Effect of DEX on Sciatic Nerve Morphology

**Figure 6:**
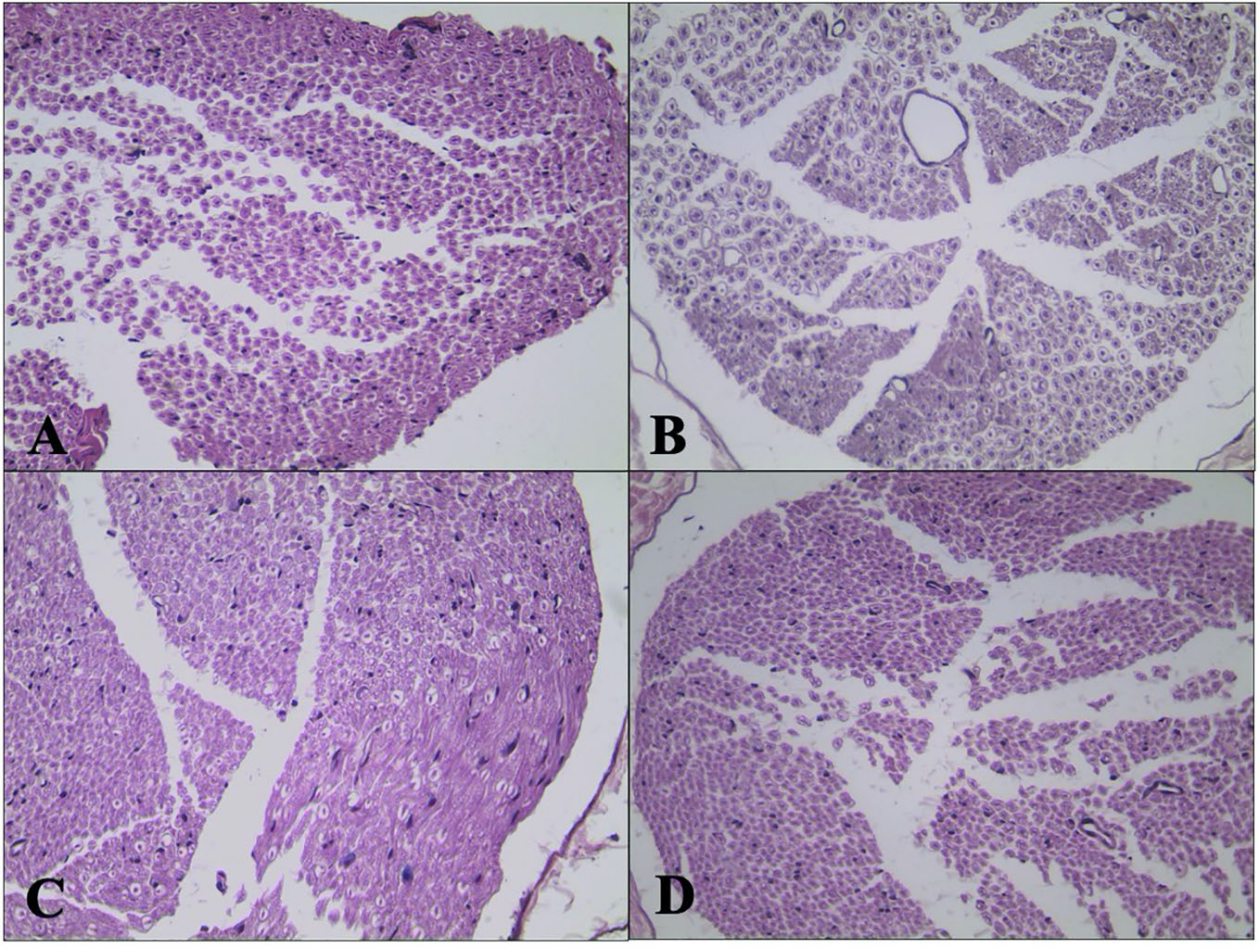
Histological examination of hematoxylin and eosin (H&E) stained sciatic nerve (n = 20). **(A)** Control **(C)** group. **(B)** Diabetic peripheral neuropathy (DPN) group. **(C)** Dexmedetomidine (DEX) group. **(D)** Yohimbine (YOH) group.

H&E staining of sciatic nerve showed normal structure in the C group (**Figure 6A**). Mild atrophy and mutation of axons and mild demyelination and myelin dissolution were shown in the DPN group (**Figure 6B**). The morphological change of sciatic nerve morphology in the DEX group (**Figure 6C**) was smaller than that in the DPN group. For the rats in the YOH group (**Figure 6D**), the morphological change was similar to that in the DPN group.

### Expression of Bax, Bcl-2, and Caspase-3 in Immunohistochemical Staining

**Figure 7:**
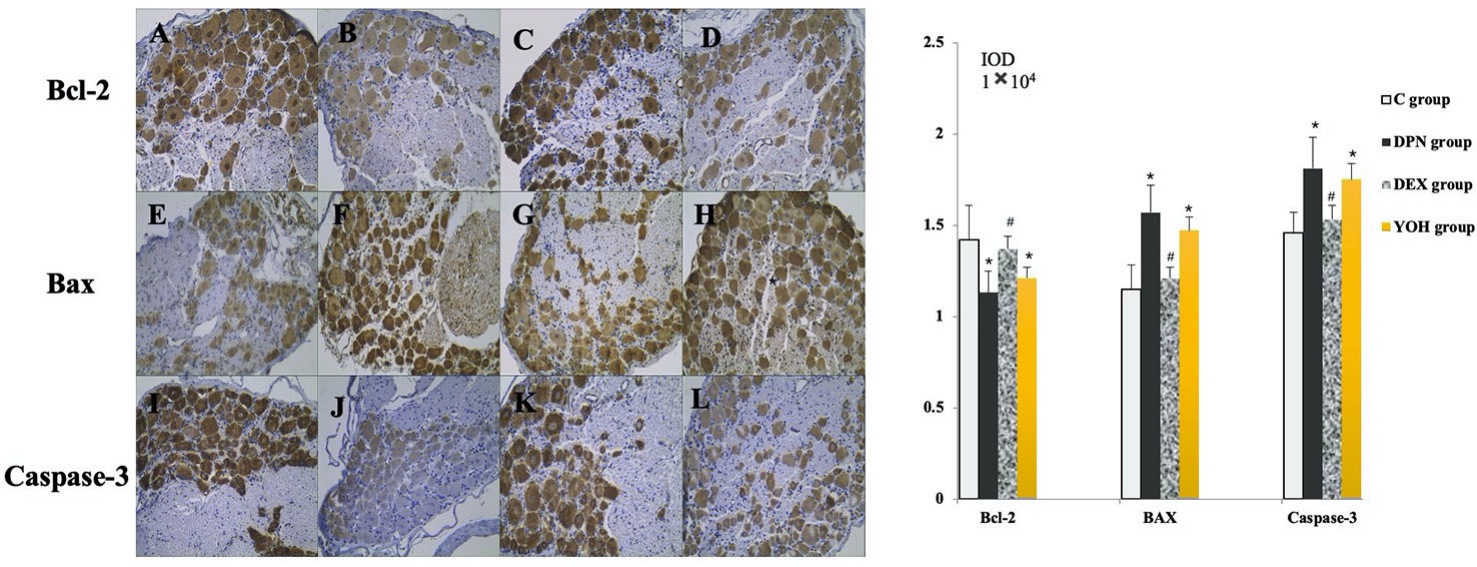
The expression of Bcl-2, Bax, and caspase-3 in immunohistochemical staining [n = 20, *p < 0.05 compared with the control (C) group; ^#^p < 0.05 compared with diabetic peripheral neuropathy (DPN) group]. **(A, E, J)** C group, **(B, F, J)** DPN group, **(C, G, K)** DEX group, **(D, H, L)** YOH group.

As shown in **Figure 7**, the expression levels of Bax and caspase-3 were increased, while that of Bcl-2 was decreased significantly in the DPN group compared with the C group (p < 0.05). In the DEX group, the expression levels of Bax and caspase-3 were decreased significantly (p < 0.05), while that of Bcl-2 was increased significantly (p < 0.05) compared with the DPN group and the YOH group.

### Western Blot Analysis of Bax/Bcl-2 Ratio and Caspase-3

**Figure 8:**
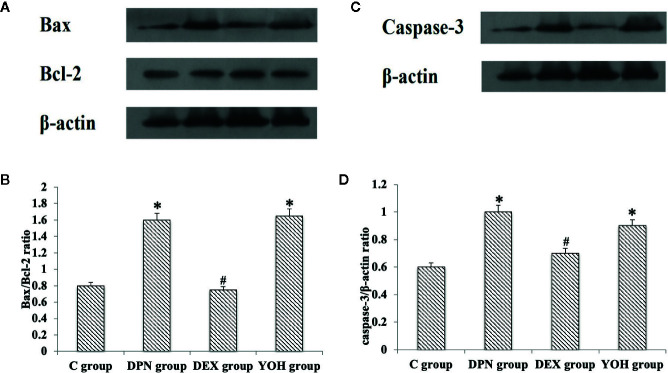
**(A, C)** is the protein expression stripe of the Bax/Bcl-2 and Caspase-3 in the western blot. **(B, D)** is the bar chart based on the grayscale of the Western stripe. [n = 20, *p < 0.05 compared with the control **(C)** group; ^#^p < 0.05 compared with DPN group].

The western blot showed that the Bax/Bcl-2 protein ratio and caspase-3 protein increased significantly in the DPN group and YOH group compared with those in the C group (p < 0.05). However, the increased Bax/Bcl-2 ratio and caspase-3 were not observed in the DEX group compared with those in the C group (**Figure 8**).

### The Expressions of Bcl-2 mRNA and Caspase-3 mRNA in Each Group

The mRNA of Bcl-2 was decreased significantly but that of caspase-3 was increased significantly in the DPN group compared with that in the C group (p < 0.05). However, in the DEX group, the mRNA of Bcl-2 was increased significantly, while that of caspase-3 was decreased significantly compared with the DPN group (p < 0.05). And the YOH group reverses this effect (**Table 3**).

**Table 3:** The expression of Bcl-2 mRNA and caspase-3 mRNA (2−ΔΔCT) in each group (n=20).

mRNA expression	C group	DPN group	DEX group	YOH group
Bcl-2	0.98±0.05	0.37±0.03*	0.71±0.04*^#^	0.41±0.06*
Caspase-3	1.01±0.04	2.73±0.12*	1.21±0.07*^#^	2.01±0.10*

C, control; DPN, diabetic peripheral neuropathy; DEX, dexmedetomidine; YOH, yohimbine.

*p < 0.05 compared with the C group, ^#^p < 0.05 compared with the DPN group.

## Discussion

DPN is the most common complication of diabetes. Therefore, it is a major scientific challenge to explore the pathogenesis of DPN and explore new treatment options. The results of this study indicated that DEX has an attenuating effect on abnormal behavior tests (MWT and TWL) induced by high glucose, low sciatic nerve conduction velocity, and cell damage in the DPN rats. In addition, the results indicated that the DEX increased the values of SOD and GSH-Px, and decreased the values of MDA and the expressions of Bax/Bcl-2 ratio and caspase-3. Finally, oxidative stress and apoptosis induced by hyperglycemia were inhibited. Yohimbine is a selective antagonist that blocks α_2_-adrenoceptor excitation of DEX, in the YOH group, the neuroprotective effect, anti-oxidative stress, and anti-apoptotic effect of DEX was reversed, which further confirmed that the neuroprotective effects attributed to DEX.

DPN and neuropathic pain is a devastating chronic condition and a common complication of diabetes (Vinik, 2005; Harati, 2007; Edwards et al., 2008). In this study, the blood glucose of the rats in the DPN group, DEX group and YOH group was always higher than 16.7 mmol/l after the STZ injection. And at the end of the study (week 10), the body weights of the rats in the DPN group, DEX group, and YOH group were significantly lower than those in the group C (p < 0.05).This showed that the diabetic models were built successfully.

And the significant differences of behavioral tests (MWT and TWL), sciatic nerve conduction velocity, and nerve histological change between the DPN group and the C group showed that the DPN model of the rats were built successfully. This study demonstrated that SD rats with STZ treatment develops DPN by the hyperglycemia. Intraperitoneal injection of STZ caused the damage of the motor as well as sensory fibers, which will result in central sensitization of neurons and reduction in the MWT and TWL. The decreased MWT and TWL in the DPN group and its amelioration were observed in the DEX treated group. Those findings are in accordance with the previous study (Lu et al., 2017). In diabetic rats, chronic hyperglycemic causes endo-neural hypoxia, which will result in oxidation of proteins and lipid of neurons, leading to reduced threshold and motor and sensory nerve conduction velocity.

Some studies have revealed that DEX has central neural protective effect (Sifringer et al., 2015; Cheng et al., 2018; Li et al., 2018). In our study, DEX increased MWT and TWL, prolonged the sciatic nerve conduction velocity and ameliorated the nerve cell damage significantly compared with the C group (p < 0.05). And the α_2_-receptors agonist yohimbine can reverse the effects of the DEX, which supported the protective effect of DEX in the DPN rats. DPN is a common considerable morbidity of the DM. The natural history of DPN suggests a progressive functional change. Ultimately, these metabolic alterations over time can lead to structural changes, including mitochondrial damage and neurodegeneration, which can be very difficult to treat or reverse (Joyce et al., 2010; Stanzione and Tropepi, 2011). In this study, Dex relieved the atrophy and mutation of axons and demyelination and myelin dissolution of the sciatic nerve. All of above showed administration of DEX exhibited the therapeutic effect on the function and structure of the nerve on type 2 diabetes.

In recent studies, oxidative stress was assessed with serum SOD, GSH-Px, and MDA in the sciatic nerve of the diabetic rats (Wu et al., 2012; Kumar et al., 2013). Oxidative stress is owe to hyperglycemia and is considered as initiating factor in the pathogenesis of DPN (Figueroa-Romero et al., 2008; Kasznicki et al., 2012). Oxidative stress will result in metabolic dysfunction of nerve fibers (Tiong et al., 2019). The oxidative stress will be increased when the lipid peroxide over-product and/or the effectiveness of antioxidant defenses is decreased. In our study, serum SOD and GSH-Px were decreased, while serum MDA was increased significantly in the DPN group compared with those in the C group (p < 0.01). SOD catalyzes the oxidation/reduction/conversion of superoxide radicals to molecular oxygen and hydrogen peroxide. GSH-Px catalyzes the hydrogen peroxide reduction by two molecules of glutathione, a part of reactive oxygen species defense system (Rao et al., 2014; Mittal et al., 2018). High glucose causes oxidative stress in different cell types. However, the production of superoxide due to the hyperglycemia is produced primarily through the mitochondrial electron transport chain, which leads to excessive production of free radicals and oxidative stress (Little and O’Brian, 1968). Ischemia and hypoxia caused by hyperglycemia leads to increased formation of reactive oxygen species (ROS), which, through oxidative stress, damage the nucleic acid, protein, lipid, and other macromolecules in nerve cells, thereby causing nerve cell damage. DEX can enhance the activity of SOD and GSH-PX, reduce the activity of MDA, improve lipid peroxidation, and inhibit the nerve injury of DPN. That may be one of the protective mechanisms of DEX against DPN.

Oxidative stress can activate some apoptosis signaling pathways such as Bax, Bcl-2, and caspase-3 (Allen and Tresini, 2000; Neamatallah et al., 2018). Increases in the levels of apoptosis related genes have been shown in the nerve tissue from animal model of DM (Venkatesan and Mohamed Sadiq, 2017; Zhu et al., 2018). Additionally, some research found that a microglial TLR4 of the spinal cord is the key receptor to initiate microglial activation and cause chronic pain after peripheral nerve injury (Tanga et al., 2005; Bettoni et al., 2008; Lu et al., 2017). Mitochondrial apoptosis is regulated by proteins that directly or indirectly activate or inhibit cysteine proteases. Bax pro-apoptotic protein directly inhibits the anti-apoptotic effect of Bcl-2 protein and prevents the formation of Bax homopolymer oligomer. Protein Bax promotes mitochondrial membrane permeability and allows the release of cytochrome c, which activates caspase-3 and caspase-9, leading to cell apoptosis (Little and O’Brian, 1968; He et al., 2018). Some studies have shown that the development of DPN includes the increase of oxidative stress, activation of cyclooxygenase, decrease of Na-K-ATPase activity, and apoptosis of nerve cell (Springer et al., 1997; Allen et al., 2005; Kumar et al., 2005). By activating 2 adrenergic receptors, DEX rectify inward potassium channels, causes intracellular K+ outflow and inhibits extracellular Ca+ inflow, which leads to hypertransformation of cell membrane, inhibition of noradrenaline activity and produce neuroprotective effect. In our study, the Bax/Bcl-2 protein ratio, caspase-3 protein, and caspase-3 mRNA were increased significantly and Bcl-2 mRNA was decreased in the DPN group compared with that in the C group (p < 0.05). That confirmed the apoptosis effect of the Bax/Bcl-2 ratio and caspase-3 in the DPN rats.

Indeed, the simultaneous production of superoxide and nitric oxide contributes to the production of peroxynitrite, which can rapidly cause the nitrification or nitrite of proteins for various cell types, lipid peroxidation, DNA damage, and cell apoptosis, and produce direct toxic effects on nerve tissues leading to neuropathy (Tang et al., 2019; Zhou and Zhang, 2019). In addition, apoptosis induced by caspase-3 activation due to oxidative stress plays an important role in the progression of diabetic neuropathy (Kamboj et al., 2010). In our study, DEX treatment up-regulated the expression of serum SOD and GSH-Px, and down-regulated the expression of serum MDA, Bax/Bcl-2, and caspase-3. These stress sensitive and neuropathic factors may also be involved in the mechanism of diabetic neuropathy. The protective effect of DEX to the DPN may be related to its anti-oxidative stress and anti-apoptotic effects.

## Conclusion

In our study, DEX can effectively prevent or alleviate DPN-related behaviors and nerve cell damage in the diabetic rats. The protective effect of DEX may result from its anti-oxidative and anti-apoptosis responses.

## Data Availability Statement

The raw data supporting the conclusions of this article will be made available by the authors, without undue reservation.

## Ethics Statement

The animal study was reviewed and approved by the ethics committee of Second affiliated hospital of Harbin Medical University.

## Author Contributions

Y-ZZ conceived the research. Y-ZZ and Z-CZ performed the research. XC and C-YS analyzed the data. Y-ZZ and XC wrote the manuscript. Y-ZZ, Z-CZ, C-YS, and XC revised the manuscript. All authors contributed to the article and approved the submitted version.

## Funding

This work was supported by the grant Natural Science Foundation of Heilongjiang province. The grant number is H2017019. The fund was provided by the Science and Technology Department of Heilongjiang Province to support basic research in the field of natural science.

## Conflict of Interest

The authors declare that the research was conducted in the absence of any commercial or financial relationships that could be construed as a potential conflict of interest.
